# The effect of rs2910686 on ERAP2 expression in IBD and epithelial inflammatory response

**DOI:** 10.1186/s12967-024-05532-w

**Published:** 2024-08-09

**Authors:** Siri Sæterstad, Ann Elisabeth Østvik, Marianne Doré Hansen, Torunn Bruland, Atle van Beelen Granlund

**Affiliations:** 1 Department of Clinical and Molecular Medicine, Norwegian University of Science and Technology (NTNU), Trondheim, Norway; 2 Department of Gastroenterology and Hepatology, Clinic of Medicine, St. Olav’s University Hospital, Trondheim, Norway; 3 Clinic of Laboratory Medicine, St. Olav’s University Hospital, Trondheim, Norway; 4 Department of Pathology, St. Olav’s University Hospital, Trondheim, Norway; 5 Centre of Molecular Inflammation Research, Norwegian University of Science and Technology (NTNU), Trondheim, Norway

**Keywords:** Inflammatory bowel disease, Inflammation, ERAP2, SNP, Colonoids, Organoids

## Abstract

**Background:**

ERAP2 is an aminopeptidase involved in antigen processing and presentation, and harbor genetic variants linked to several inflammatory diseases such as Inflammatory Bowel Disease (IBD). The lack of an ERAP2 gene homologue in mice has hampered functional studies, and most human studies have focused on cells of hematopoietic origin. Using an IBD biobank as vantage point, this study explores how genetic variation in ERAP2 affects gene expression in human-derived epithelial organoids upon proinflammatory stimulation.

**Methods:**

An IBD patient cohort was genotyped with regards to two single nucleotide polymorphisms (SNP) (rs2910686/rs2248374) associated with ERAP2 expression levels, and we examined the correlation between colon gene expression and genotype, specifically aiming to establish a relationship with ERAP2 expression proficiency. Human-derived colon organoids (colonoids) with known ERAP2 genotype were established and used to explore differences in whole genome gene expression between ERAP2-deficient (*n* = 4) and -proficient (*n* = 4) donors upon pro-inflammatory encounter.

**Results:**

When taking rs2910686 genotype into account, ERAP2 gene expression is upregulated in the inflamed colon of IBD patients. Colonoids upregulate ERAP2 upon IFNɣ stimulation, and ERAP2 expression proficiency is dependent on rs2910686 genotype. Colonoid genotyping confirms that mechanisms independent of the frequently studied SNP rs2248374 can cause ERAP2-deficiency. A total of 586 genes involved in various molecular mechanisms are differentially expressed between ERAP2 proficient- and deficient colonoids upon proinflammatory stimulation, including genes encoding proteins with the following molecular function: catalytic activity (*AOC1*, *CPE*, *ANPEP* and *MEP1A*), regulator activity *(TNFSF9*, *MDK*, *GDF15*, *ILR6A*, *LGALS3* and *FLNA*), transmembrane transporter activity *(SLC40A1* and *SLC5A1*), and extracellular matrix structural constituents (*FGL2*, *HMCN2*, and *MUC17*).

**Conclusions:**

ERAP2 is upregulated in the inflamed IBD colon mucosa, and expression proficiency is highly correlated with genotype of rs2910686. While the SNP rs2248374 is commonly used to determine ERAP2 expressional proficiency, our data confirms that mechanisms independent of this SNP can lead to ERAP2 deficiency. Our data demonstrates that epithelial ERAP2 presence affects the inflammatory response in colonoids, suggesting a pleiotropic role of ERAP2 beyond MHC class I antigen processing.

**Supplementary Information:**

The online version contains supplementary material available at 10.1186/s12967-024-05532-w.

## Background

Most immune-mediated diseases show some heritability, and over the last few decades many studies have identified specific genetic variants linked to disease risk. While this has increased our understanding of cellular mechanisms involved in disease development, only a handful of risk variants have been functionally characterized [1]. Inflammatory bowel disease (IBD) has been extensively studied, resulting in the identification of more than 240 IBD risk loci, many of which are shared with other immune mediated diseases [2]. In this study we explore one of these genes, Endoplasmic reticulum aminopeptidase 2 (ERAP2), using human-derived colonoids carrying relevant genetic variants as model.

ERAP2 is primarily known for trimming of peptides to be presented by major histocompatibility class I (MHCI) molecules. ERAP2 shares ∼ 49% sequence homology with ERAP1, and they both belong to the oxytocinase subfamily of M1 aminopeptidases [3]. There is some redundancy between ERAP1 and ERAP2 with regards to MHCI peptide processing, but the two proteins differ from each other in both peptide length preference and N-terminal amino acid cleaving efficiency [3–5]. The ERAP1/ERAP2 ratio is thus proposed to impact the peptide repertoire presented through MHCI, and thereby the following immune response.

Single nucleotide polymorphism (SNP) rs2248374 (A/G) is a well-established determinant of ERAP2 expression. The A allele is thought to give rise to functional protein, whereas the G allele results in a truncated mRNA degraded through nonsense-mediated decay. Due to balancing selection of rs2248374, about one in four individuals are G/G at this locus globally and will hence be devoid of full length ERAP2 protein expression [6]. As multiple isoforms can be transcribed from ERAP2, we want to emphasize that, in this article, ERAP2-deficiency specifically refers to the absence of the full-length ERAP2. Deficiency in 25% of the population implies that the full-length protein is dispensable for a functional MHCI presentation, and ERAP2 has accordingly been viewed as an accessory aminopeptidase in antigen presentation. In addition, several species (including rodents) lack the ERAP2 gene, and the biological significance and function of ERAP2 has thus been less extensively studied compared to its homolog ERAP1.

Selection for functional ERAP2 is thought to have arisen as a protective mechanism towards infection [7, 8]. However, increased ERAP2 expression is in general associated with risk of chronic inflammatory diseases, including IBD (especially Crohn’s disease), ankylosing spondylitis, birdshot chorioretinopathy, psoriasis, preeclampsia, and hypertension [9–11]. Thus, ERAP2 expression seems to have opposing outcomes depending on environmental and biological states.

Additional functions of ERAP2 beyond MHCI peptide-trimming have emerged in recent years, spanning from antiviral properties and modulation of pyroptosis and autophagy, to involvement in the renin-angiotensin system [5, 12–14]. Moreover, it has been shown that ERAP2 can interact with both Insulin-Regulated Aminopeptidase (IRAP) and Epithelial Cell Adhesion Molecule (EpCAM), and that a short isoform of ERAP2 (Iso3) is induced and secreted upon microbial stimuli [15–17]. Most of these observations are demonstrated in immune cells, while little is known about possible pleiotropic effects of epithelial ERAP2. The intestinal epithelium is our first line defense against external factors encountered in the lumen, acting both as a physical and immunological barrier. Most infectious agents invade the body through mucosal surfaces, emphasizing the importance of a fully functional and immunologically active epithelial barrier.

IBD is an umbrella term encompassing ulcerative colitis (UC) and Crohn’s disease (CD), conditions involving inflammation of the gastrointestinal tract [18]. As a multifactorial disease, IBD arises through an interplay of genetics, environment, and the microbiome. Hallmarks of IBD include dysregulation of microbial sensing, autophagy, and loss of intestinal homeostasis, all involving the epithelial barrier [19]. GWAS studies have linked genetic variants in ERAP2 to increased IBD risk, but little is known regarding ERAP2 expression pattern and function in active IBD [20]. Most previous studies of ERAP2 have revolved around immune cells, whereas the epithelial effect of varying ERAP2 expression is relatively unexplored.

In this study we have explored the expression of ERAP2 in IBD, and how colon epithelial ERAP2 expression is affected by genotype. We show that ERAP2 is upregulated in the colon during active IBD. By studying human-derived ERAP2-deficient vs. ERAP2-proficient 3D primary epithelial cell cultures (colonoids) upon proinflammatory stimulation, we shed light upon epithelial cellular pathways that are potentially affected by ERAP2 presence under inflammatory conditions. We also confirm that mechanisms independent of rs2248374 affect the protein expression levels of ERAP2.

## Methods

### Clinical material

All subjects were recruited when admitted to St. Olav’s University Hospital for colonoscopy. The study was approved by Central Norway Regional Committee for Medical and Health Research Ethics (2013/212/REKmidt, #134436). The participants provided their written informed consent to participate in this study. Inflamed (*n* = 37) samples were taken from maximally inflamed colonic mucosa with intact epithelium. Uninflamed samples (*n* = 36) were all taken from the hepatic flexure. The total number of enrolled individuals is lower than the number of samples, as paired samples (inflamed/uninflamed) from 21 patients were included. Blood samples were drawn from all individuals included, and subsequently used for DNA isolation. Characteristics of IBD patients included in genotype and gene expression correlation is presented in Table 1. Four closely adjacent biopsies were taken from each area. Two were immediately snap frozen and stored in liquid N2 for subsequent RNA isolation, while the two remaining were formalin fixed for subsequent histological confirmation of disease activity. Inflammatory status was verified by inspection of hematoxylin and eosin-stained sections and classified into healthy, uninflamed, or active inflammation by an experienced pathologist.

**Table 1:** Characteristics of IBD patients included in genotype and gene expression correlation

	Active IBD (*n* = 37)	Inactive IBD (*n* = 36)
Age	37 (18–76)	41 (18–76)
Sex (F)	20 (54%)	20 (55%)
5-ASA	18 (49%)	20 (55%)
Steroids	16 (43%)	14 (39%)
Anti-TNFα	4 (11%)	2 (5%)
UC	20 (54%)	19 (53%)
CD	17 (46%)	16 (44%)

*IBD* Inflammatory bowel disease, *5-ASA* 5-aminocalicylic acid, *TNFα* Tumor necrosis factor alpha, *UC* Ulcerative colitis, *CD* Crohn’s disease

Organoids were selected from an in-house biobank based on genotyping status. Patient material used for organoid establishment was taken from endoscopically intact colonic mucosa, both from IBD patients and non-IBD controls, and immediately used in organoid establishment as described later.

### Nucleic acid isolation

Total RNA was extracted from snap frozen colonic pinch biopsies and colonoids using the Ambion mirVana RNA extraction kit (Applied Biosystems), according to the manufacturers protocol. Biopsies were transferred to lysis buffer and homogenized using a T10 Ultra Turrax homogenizer (IKA, Fisher Scientific, USA). For colonoids, media was first removed, and colonoid-matrigel domes were gently rinsed with PBS (37 °C) before placing the plate on ice. Domes were lysed directly in the culture plate (275µL lysis buffer/well, 24well-plate) and collected in 1.5 mL tubes. Three wells were combined for each condition. After vortexing, the samples were stored at -80 °C until RNA isolation. RNA was extracted from lysates using a *mir*Vana™ miRNA Isolation Kit (AM1560, Invitrogen), following the manufacturers protocol for total RNA isolation. RNA quality and concentration was determined using Bioanalyzer (Agilent Technologies, Santa Clara, CA, USA) and Qubit or Nanodrop (Thermo Fisher Scientific Inc., Waltham, MA, USA). All isolated RNA samples were stored at − 80^o^C or below until analysis. DNA was isolated from either EDTA whole blood or clotted blood. For clotted blood samples, a modified version of the MasterPure DNA Purification kit Blood Version II (Epicentre, Illumina, USA) was used. In brief, whole blood clots were homogenized (full speed, 20 s) using a T10 Ultra Turrax homogenizer (IKA, Fisher Scientific, USA), and dissolved in 15 mL Red Cell lysis buffer. After incubation on a rocking shaker (60 rev/min, 5 min), the lysed material was pelleted (3 min, 1900 rpm, 18^o^C) and supernatant removed. The pellet was then reconstituted in Red Cell Lysis (10 mL), and the process of shaking and centrifugation was repeated twice. After three rounds of lysis, the supernatant was removed, and pellet dissolved by quickly vortexing in Tissue and Cell-lysis solution (5 mL) and Proteinase K (25 µl) and left to incubate (2 h, 55^o^C). After incubation, MPC Protein Precipitation solution (3 mL) was added, and sample was vortexed for 20 s, followed by centrifugation (10 min, 3400 rpm, 10^o^C) to pellet the precipitated proteins. Supernatant was transferred to a tube containing isopropanol (10 mL) and mixed by inverting 30–40 times. DNA was then pelleted through centrifugation (2 min, 3000 rpm, 18^o^C), and supernatant was removed. The isolated DNA was subsequently washed by adding 70% ethanol (5 mL), followed by centrifugation (1 min, 3000 rpm, 18^o^C). Supernatant was decanted, centrifugation repeated, and sample left under fume hood to remove last traces of ethanol. As a final step, the DNA was resuspended in TE buffer (500 µL), incubated (2 h, 65^o^C) and left in room temperature overnight, followed by shaking to ensure complete dissolvement of DNA pellet in TE buffer. For isolation of DNA from EDTA full blood, a Chemagic Star nucleic acid extraction robot (Hamilton Robotics, PerkinElmer, USA) was used, in combination with chemagic STAR DNA Blood400 Kit (Chemagen, PerkinElmer, USA). The isolation followed standard protocol, extracting DNA from 400 µl EDTA full blood, and eluting in 150 µl elution buffer. All DNA samples were stored at -80^o^C prior to analysis.

### Genotyping and correlation to gene expression

Patient genotyping data was extracted from a large in-house data set of genotyped IBD patients. Patient samples were genotyped using Illumina iSelect InfiniumCoreExome genotyping arrays, containing 260k GWAS markers and 260k exome variants. Additional genotypes were identified using the Michigan Imputation Server [21]. Final dataset only includes individuals for whom both genotype and gene expression data were available. Furthermore, only genotyping status of the SNPs rs2910686 and rs2248374 was used in the subsequent analysis. Normalized ERAP2 gene expression data was also extracted from an in-house RNA-sequencing (RNA-Seq) dataset of colon gene expression, of which all included individuals were genotyped for the two SNPs in question. RNA-seq analysis was performed as described in the paragraph “RNA-sequencing”. Data were used to explore the correlation between gene expression levels and genotype of ERAP2, as well as to investigate the gene expression levels of ERAP2 during colonic inflammation when considering genotype.

SNP genotyping of organoid donors (input DNA 10ng) was performed on a StepOnePlus™ Real-Time PCR System (Applied Biosystems), using TaqMan™ Genotyping Master Mix (#4371355, Applied Biosystems) and predesigned TaqMan^®^ SNP Genotyping Assays (Applied Biosystems) for rs2910686 (C_26382310_10) and rs2248374 (C_25649529_10). Experiments were performed following the manufacturers protocol for wet DNA.

### Colonoid culture, media and experiments

**Fig. 1:**
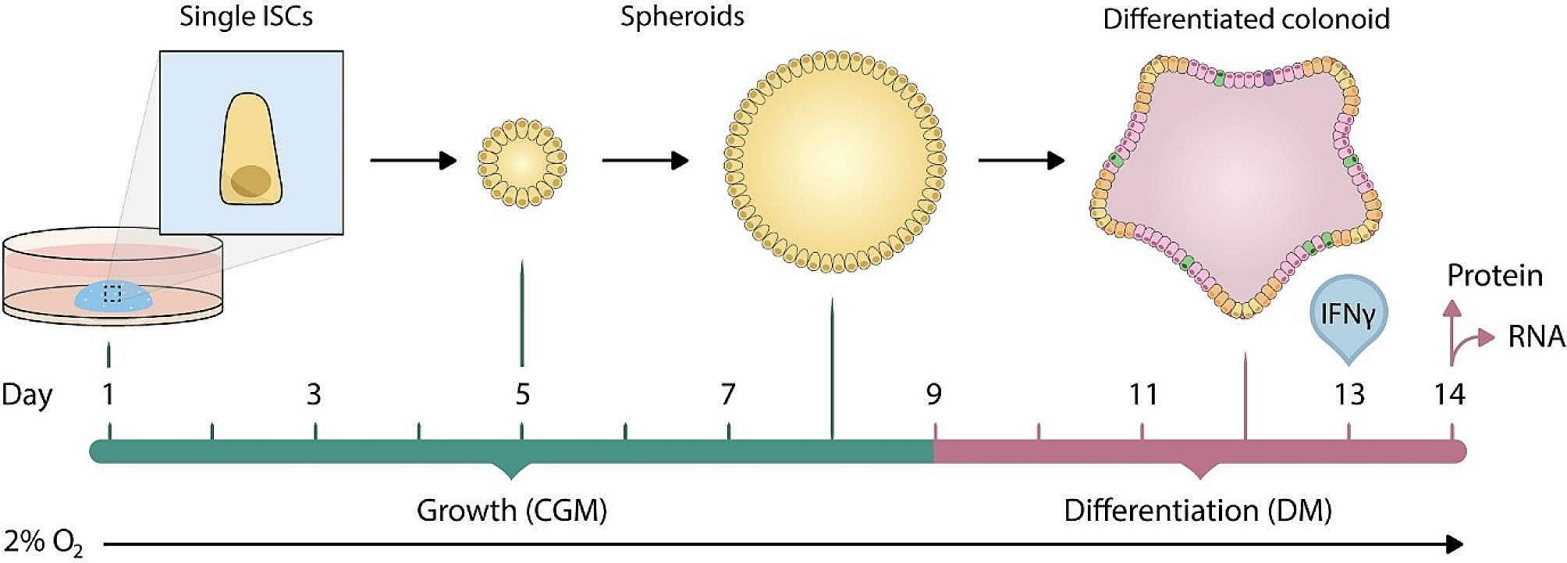
Experimental timeline for all colonoid-donors included in the study. On day one, intestinal stem cells (ISCs) were resuspended in Matrigel and seeded into 24-well plates. From day one through day eight, colonoids were grown in complete growth medium to support stem cell growth. Prior to differentiation, colonoids appear spheric (spheroids) and are mostly composed of immature cells. From day nine throughout the experiments, colonoids were grown in differentiation media to induce epithelial maturation. On day 13, colonoids were stimulated with IFNγ (10ng/mL). On day 14, colonoids were harvested for RNA and protein isolation. To better mimic in vivo physiological oxygen levels, colonoids were grown at 2% O_2_

Human colonoids were established from colonic biopsies, based on protocols of Mahe et al. and Jung et al. [22, 23]. Composition of complete growth medium (CGM) and differentiation medium (DM) is listed in Additional File 1: Table S1-S3. Detailed protocols for colonoid establishment and media production are described in Gopalakrishnan, S. et al. [24]. Expansion cultures were grown in CGM for seven days before passaging. For all experiments, intestinal stem cells were resuspended in Matrigel (#356231, Corning, 7.3–8.1 mg/mL protein) to a final concentration of 160-200cells/µL, resulting in 8000-10 000 cells/well. Cell-Matrigel suspension was plated on pre-warmed 24w plates (50µL/well) and incubated at 37 °C for 20 min prior to addition of 500µL CGM. On day one and three, CGM was supplemented with Y-27,632 (#72304, STEMCELL Technologies, 3.203 µg/mL) to prevent anoikis. CGM supports stem cell growth. From day nine and throughout the experiment, colonoids were grown in DM to induce differentiation. On day 13, colonoids were stimulated with IFNγ (#300-02, PeproTech, 10ng/mL). The day of stimulation, A-83-01 was withdrawn from the DM. Two experimental plates for each donor were run in parallel, one for RNA extraction (three wells/condition) and one for protein isolation (four wells/condition). Colonoids were harvested 24 h post stimulation. As 2% oxygen, rather than the traditional 20%, better resembles the in vivo oxygen level of the colonic mucosa, colonoids were grown in 2% O_2_ and 5% CO_2_ throughout all experiments [25]. Media was refreshed every two to three days. Experimental timeline is visualized in Fig. 1. Table 2 provides characteristics of ERAP2-proficient and -deficient donors.

For initial experiments, ERAP2 protein expression under three different conditions was tested in one proficient and one deficient donor: proinflammatory cocktail, IFNγ (10ng/mL, #300-02, PeproTech) and IFNα (10ng/mL, #SRP4596, Sigma-Aldrich). The proinflammatory cocktail included TNFα (#300–01 A), IFNγ, IL1β (#200-01B), IL17α (#200 − 17), IL22 (#200 − 22) all from PeproTech, and Poly I: C (Invivogen). In exception of Poly I: C (0.63 µg/mL), the concentration for all ligands in the cocktail were 3.13ng/mL.

**Table 2:** Characteristics of ERAP2-proficient and -deficient donors included in the study

	Proficient	Deficient
Donors	4	4
Median age (range)	23.5 (18-55)	32 (20-48)
Females	2	3
IBD	3	1
5-ASA/S-ASA	2	1
Steroids	2	0
Anti-TNFα	1	0
Imurel	2	0
Biopsy location	HF (2), LF (2)	HF (4)

*IBD* Inflammatory bowel disease, *5-ASA* 5-aminocalicylic acid, *S-ASA* Sulphasalazine, *TNFα* Tumor necrosis factor alpha, *HF* Hepatic flexure, *LC* Left colon

### cDNA synthesis and qPCR

Reverse transcription was performed using a High-Capacity RNA-to-cDNA™ Kit (#4387406, Applied Biosystems). TaqMan real-time qPCR assays were performed on cDNA samples using TaqMan Gene Expression Assay for ERAP2 (Hs01073631_m1), normalized to ACTB (Hs01060665_g1). Relative gene expression was calculated using the Delta-Delta Ct Method.

### RNA-Sequencing

RNA-Seq was performed in the same way for both patient biopsies and colonoid isolates. For colonoid-RNA, three wells were combined for each condition. RNA concentration was measured using Qubit^®^ RNA HS Assay Kit on a Qubit^®^ 3.0 Fluorometer (Thermo Fisher Scientific Inc., Waltham, MA, USA). Integrity was assessed using Agilent RNA 6000 Nano Kit on a 2100 Bioanalyzer instrument (Agilent Technologies, Santa Clara, CA, USA).

RNA-Seq libraries were prepared using the Illumina Stranded mRNA prep (ligation) kit (Illumina, San Diego, CA, USA) according to the manufacturer’s instructions. In brief, 500 ng total RNA was used as starting material. First, mRNA was purified from the total RNA using poly-T oligo-attached magnetic beads, followed by random fragmentation at 94°C for 8 min. First strand cDNAs were synthesized (42°C for 15 min) using random hexamer oligonucleotides and includes Actinomycin D, which allows RNA-dependent synthesis and improves strand specificity while preventing spurious DNA-dependent synthesis. Second strand cDNA synthesis (16°C for 1 hour) followed by 3’ end adenylation was performed (37 °C for 30 min), and anchors ligated to facilitate a second ligation of Illumina dual index adapter oligonucleotides (30 °C for 10 min). After a fragment cleanup step using the AMPure XP (Beckman Coulter, Inc., Indianapolis, IN, USA), library fragments were enriched by 11 cycles of PCR reaction. The final libraries were purified using the AMPure XP (Beckman Coulter, Inc., Indianapolis, IN, USA), quantitated by qPCR using KAPA Library Quantification Kit (Kapa Biosystems, Inc., Wilmington, MA, USA) and validated using Agilent High Sensitivity DNA Kit on a Bioanalyzer (Agilent Technologies, Santa Clara, CA, USA). The size range of the DNA fragments were measured to be in the range of app. 200–1000 bp and peaked around 336 bp. Libraries were normalized and pooled to 2.3 pM and subjected to clustering on a NextSeq 500 HO flowcell (Illumina, San Diego, CA, USA). Finally, paired end read sequencing was performed for 2 × 148 cycles on a NextSeq 500 instrument (Illumina, Inc. San Diego, CA, USA), according to the manufacturer’s instructions. Base calling was done on the NextSeq 500 instrument by RTA 2.4.6. FASTQ files were generated using bcl2fastq2 Conversion Software v2.20.0.422 (Illumina, Inc. San Diego, CA, USA). FASTQ files quality controlled with fastqc (v0.11.9), then filtered and trimmed by fastp (v0.20.1). Trimmed sequences were aligned to the genome reference using STAR (v2.7.9a) and quality metrics were extracted with picard CollectRNASeqMetrics (v2.21.5). Transcript counts were generated using quasi alignment (Salmon v1.7.0) to the GRCh38 transcriptome reference sequences. Transcript counts were imported into the R statistical software and aggregated to gene counts using the tximport (v1.120) bioconductor package for downstream statistical analysis. Gene counts were normalized and analyzed for differential expression using the DESeq2 bioconductor package. In the case of patient biopsies, ERAP2 expression data was extracted as normalized counts, prior to any differential expression analysis.

Quality control of the colonoid RNA-seq data identified one sample (Donor X) as a biological outlier, with gene expression patterns significantly differing from what was seen across all other samples. This sample’s data was thus removed from the RNA-seq analysis and all subsequent gene and protein expression analyses.

### Western blot

Colonoids were collected 24 h post stimulation, following the procedure described in *Protocol 5* by Gopalakrishnan et al. [24]. For each condition, four wells were combined. Isolated colonoid pellets were lysed for 2 h on a shaker (4 °C) in lysis buffer composed of 50 mM Tris-HCl pH 7.5, 150 mM NaCl, 5 mM EDTA, 1% NP-40, 1 mM DTT, 1x Complete^®^ EDTA-free protease inhibitor (#11873580001, Sigma-Aldrich), and 1x Phosphatase Inhibitor Cocktail II (#P5726, Sigma-Aldrich) and III (#P0044, Sigma-Aldrich). Lysates were then centrifuged at 14 000 × g for 20 min (4 °C), and the supernatant collected. Pierce™ BCA Protein Assay Kit (#23227, Thermo Scientific) was used for determination of protein concentration. Protein loaded was either 22ug or 35ug. Proteins were denatured for 10 min at 70 °C in 1X NuPAGE™ LDS Sample Buffer (#NP007, Invitrogen) supplemented with DTT (100 mM). Separation of proteins was performed using mini and midi 4–12% NuPage Bis-Tris gels (#NP0322BOX/#WG1402BOX, Invitrogen) in 1X NuPAGE™ MOPS SDS Running Buffer (#NP0001, Invitrogen). PageRuler and PageRuler Plus (#26616/#26619, Thermo Scientific) were loaded as protein ladders (3uL). Gels were electroblotted onto 0.2 μm nitrocellulose membranes (#1704158/#1704159, BioRad) using a Trans-Blot Turbo Transfer System (Bio-Rad). The membranes were blocked in TBS-Tween (0.05%) supplemented with BSA (1%) for 1 h at room temperature before primary antibody incubation overnight (4 °C). The following day, membranes were washed 3 × 10 min in TBS-Tween (0.05%) prior to secondary antibody incubation (1 h, RT). Membranes were then washed 2 × 10 min TBS-Tween (0.05%) and 1 × 10 min in TBS before visualization in a LI-COR Odyssey. Images were analyzed using Image Studio Software (LI-COR Biosciences). Protein levels were normalized to GAPDH and are presented as fold change. Primary antibodies used: ERAP2 (#AF3830, R&D Systems, 1:2000), GAPDH (#5174S, Cell Signaling Technology, 1:5000). Secondary antibodies used: Goat anti-Rabbit 800 (#SA5-35571, Invitrogen, 1:5000), Donkey anti-Goat 647 (#A-21447, Invitrogen, 1:1000). Full blots are available in Additional File 2 and 3: Figure S1 and S2.

### Enrichment analysis

The list of differentially expressed colonoid genes (*P* < 0.05, 586 genes) in the contrast ERAP2-deficient vs. ERAP2-proficient colonoids (IFNγ-stimulated) were uploaded to MetaCore (Clarivate) for enrichment analysis in gene sets within the gene onthology (GO) catecories *Molecular Functions*, *Processes*, and *Localizations*. Grouping of thresholded genes (100 in total) was based on Metacore *Molecular Functions.*

### Statistics

To compare colonoid ERAP2 expression in response to IFNγ, IFNα and cocktail stimulation with control, a comparison of ERAP2 protein expression using western blot was performed. The experiment was repeated four times and combined as expression levels relative to housekeeping control (GAPDH). One-way ANOVA with Dunnett correction for multiple comparisons was performed to compare ERAP2 expression response between stimuli. For relative ERAP2 mRNA and protein expression comparison between organoids before and after IFNγ stimulation, a paired t-test was used, comparing each donor before and after stimulation with itself. To compare *ERAP2* expression in inflamed and noninflamed biopsies, samples were max/min normalized within each genotype to correct for genotype-effect on expression levels. After normalization, an unpaired t-test was performed. The statistical test used for gene expression analysis of RNA-seq-data is described in the “RNA-Sequencing” section.

## Results

### Patient-derived colonoids reconstitute patient-heterogeneity in an ex vivo model

**Fig. 2:**
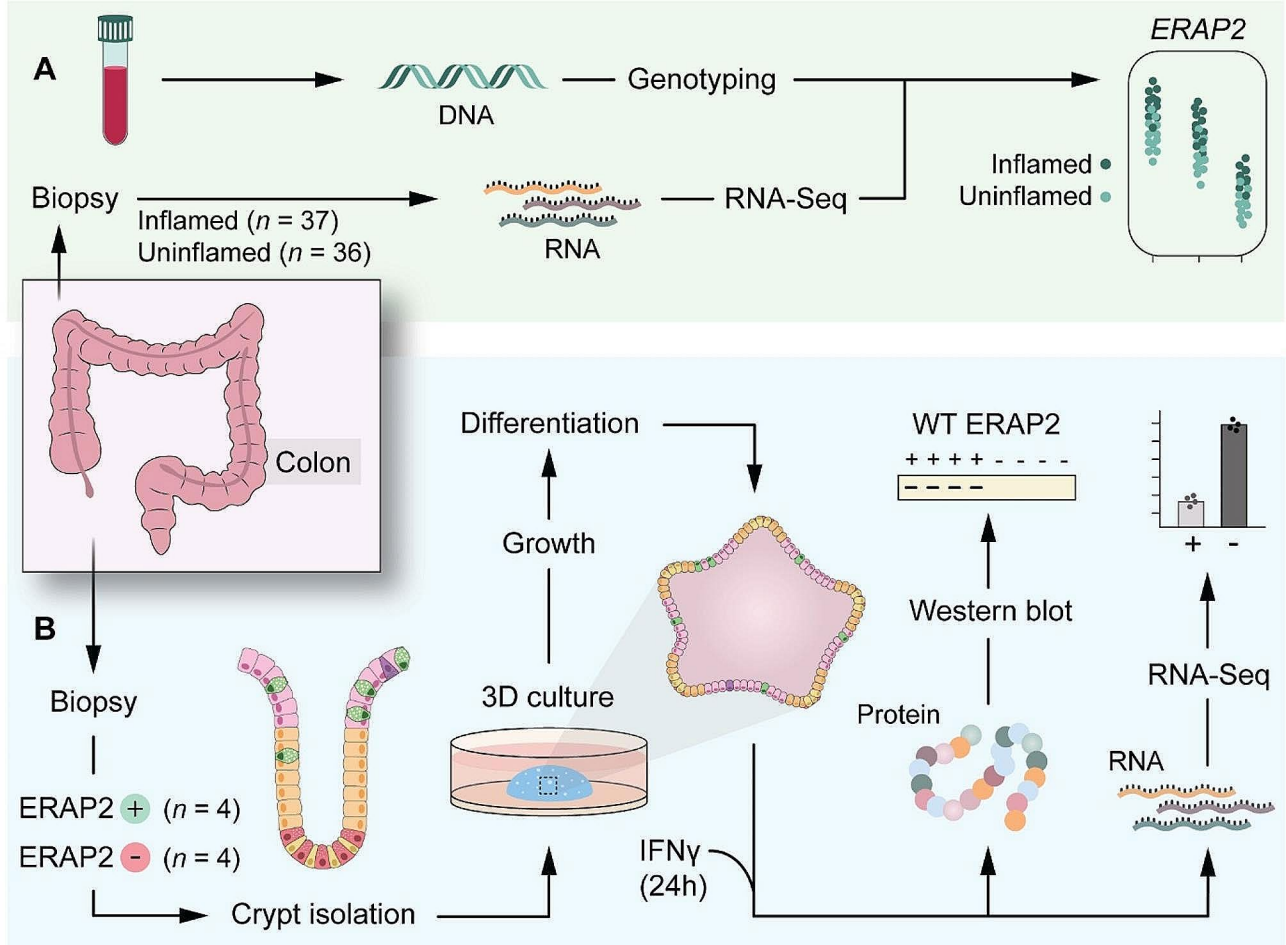
Experimental strategy for (**A**) patient *ERAP2* gene expression and genotype correlation and (**B**) human-derived colonoid verification and functional studies. (**A**) DNA was extracted from blood samples drawn from an IBD biobank and genotyped. For the same patients, RNA was extracted from colon biopsies, and *ERAP2* gene expression in inflamed and uninflamed colon mucosa was determined. *ERAP2* genotype and gene expression were correlated in order to identify SNPs affecting gene expression. (**B**) From the same IBD biobank, patient biopsies were used to establish colonoids with known *ERAP2* genotype. Differentiated colonoids were stimulated with IFNɣ (24 h) to induce an inflammatory response. Protein and mRNA extracts were used to confirm differences in ERAP2 expression proficiency dependent on genotype. RNA extracts were further sequenced, and the difference in inflammatory response of ERAP2-proficient and -deficient colonoids was characterized

Linking disease-relevant genes to functional differences is a recurrent challenge. In this study we aimed to elucidate the effect of ERAP2-proficiency and -deficiency in colon epithelium. To address this challenge, we chose to establish colonoid-cultures from a selection of ERAP2-proficient and -deficient individuals previously enrolled in a longitudinal IBD cohort. The research strategy chosen is outlined in Fig. 2.

### *ERAP2* is differentially expressed in IBD mucosa

**Fig. 3:**
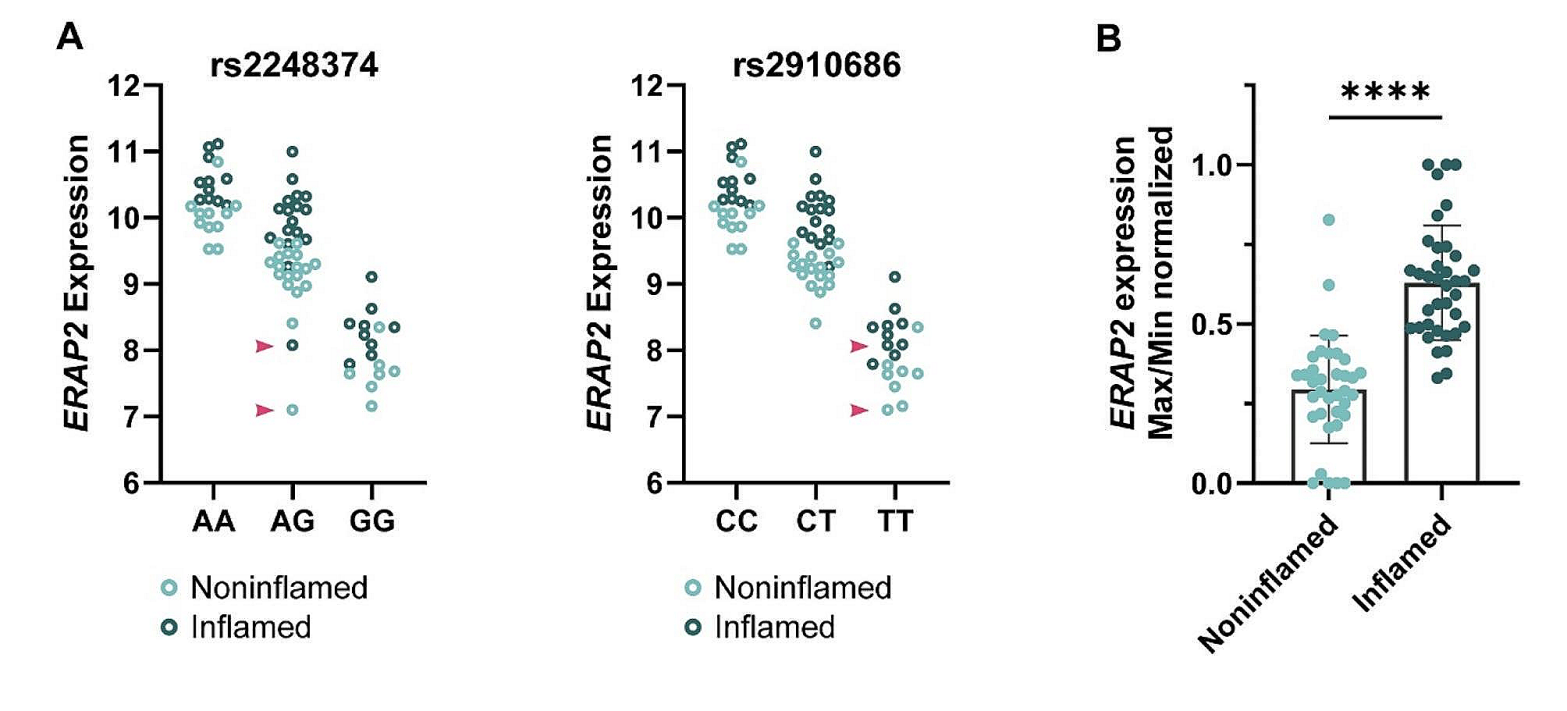
*ERAP2* expression in inflamed and noninflamed colonic pinch biopsies. **A**) *ERAP2* expression for rs2248374 (A/G) and rs2910686 (C/T) in inflamed (*n* = 37, dark green) and noninflamed (*n* = 36, light green) pinch biopsies across genotype. Expression is presented as log_2_ transformed, normalized counts. Red arrows indicate samples where rs2248374 and rs2910686 are discordant, i.e. showing different genotype than expected through linkage disequilibrium. **B**) *ERAP2* expression is increased in active IBD compared to uninflamed samples (*P* < 0.0001). Within each rs2910686 genotype, expression levels were max/min normalized to correct for genotype effect on expression levels. An unpaired t-test was used to compare noninflamed and inflamed samples

An in-house dataset of genotyped IBD patients coupled with paired gene expression data was used to evaluate *ERAP2* expression during active disease. Expression pattern across genotype for rs2248374 (A/G) and rs2910686 (C/T) is presented in Fig. 3A. When taking rs2910686 genotype into account of sequenced pinch biopsies (inflamed/noninflamed), we found an upregulation of ERAP2 during active inflammation after doing max/min normalization for all samples within each genotype (Fig. 3B).

### ERAP2 is induced in colonoids upon IFNγ stimulation

**Fig. 4:**
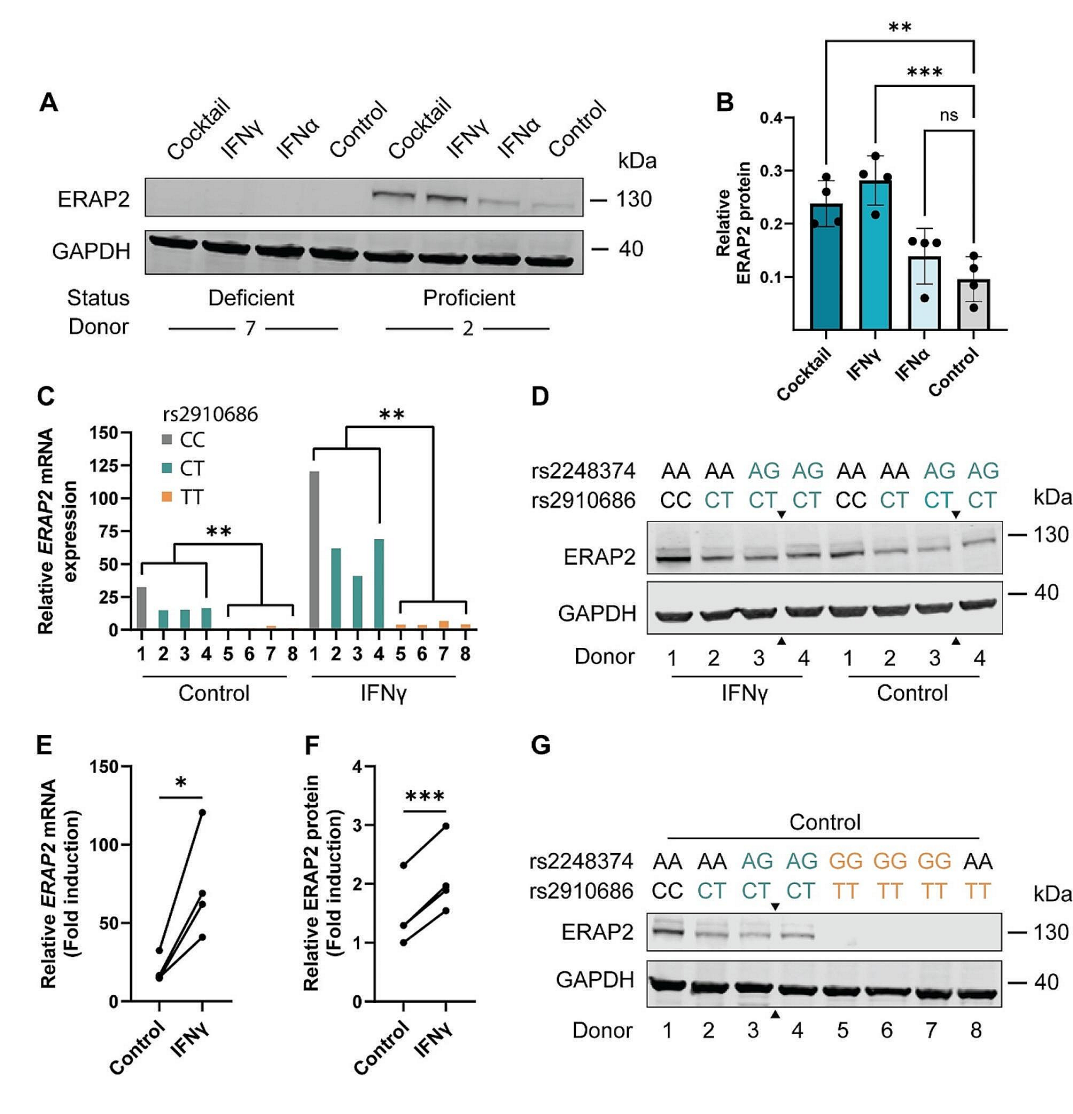
*ERAP2* mRNA and protein levels in human-derived colonoids post proinflammatory stimulation. **A**) Representative western blot showing ERAP2 expression in Donor 7 (deficient) and Donor 2 (proficient) 24 h post cocktail (TNFα, IFNγ, IL1β, IL17α, IL22 and Poly I: C), IFNɣ and IFNα stimulation. **B**) Quantification of ERAP2 protein expression in four independent experiment (Donor 2) 24 h post cocktail, IFNɣ and IFNα stimulation. Values are relative to internal control (GAPDH). A one-way ANOVA with Dunnett correction for multiple comparisons was performed. **C**) Relative *ERAP2* mRNA expression in proficient (*n* = 4) and deficient (*n* = 4) donors normalized to *ACTB* as measured with rt-qPCR. Genotype for rs2910686; CC (gray), CT (green), TT (yellow). **D**) ERAP2 protein expression in proficient donors (*n* = 4) 24 h post IFNγ stimulation and in unstimulated controls. Genotype for rs2248374 and rs2910686 indicated for each donor. **E**) Quantification of relative *ERAP2* mRNA levels in proficient donors after IFNγ stimulation. A paired t-test shows induction of *ERAP2* mRNA (*P* = 0.026) 24 h post IFNγ stimulation. **F**) Quantification of relative ERAP2 protein levels after stimulation. A paired t-test show induction of ERAP2 protein (P value < 0.001) 24 h post IFNγ stimulation. **G)** Verification of ERAP2-proficient and deficient donors. Genotype for rs2248374 and rs2910686 indicated for each donor. Donor 8 (rs2248374 AA, rs2910686 TT) does not express detectable ERAP2 protein. Figures 3G and 4D have been cropped (arrows marked), full blot available in Additional file 3: Figure S2

Initially, three conditions were tested in one ERAP2-proficient (Donor 2) and one ERAP2-deficient donor (Donor 7) to evaluate ERAP2 protein induction: (1) Proinflammatory cocktail (TNFα, IFNγ, IL1β, IL17α, IL22 and Poly I: C), (2) IFNγ and (3) IFNα (Fig. 4A). The rationale for including the inflammatory cocktail was based on in-house gene expression data for an ERAP2-proficient donor (Donor 2) showing upregulation of ERAP2 24 h post proinflammatory cocktail-stimulation. In the case of IFNα, Saulle et al. (2020) have previously shown that IFNα can induce expression of a short ERAP2 isoform (Iso3) in monocyte-derived macrophages [15]. We did not observe protein expression of this isoform in the two colonoid donors investigated, full blot provided in Additional File 2: Figure S1. ERAP2 protein expression was induced by the proinflammatory cocktail and IFNγ, whereas IFNα-treated colonoids expressed levels comparable to untreated colonoids (Fig. 4B). IFNγ is a known inducer of ERAP2 expression in various cells, albeit to our knowledge not previously investigated in human colon epithelial cells [26]. We found ERAP2 to be significantly induced at both the mRNA and protein level in colonoids 24 h post IFNγ stimulation in all ERAP2-proficient donors investigated (Fig. 4C-F).

### Mechanisms independent of rs2248374 affects expression of ERAP2

Gene expression and western blot analysis revealed that Donor 8 (rs2248374 AA/rs2910686 TT), was ERAP2-deficient. Donor 8 displayed mRNA expression levels equal to rs2248374 GG/rs2910686 TT individuals (Fig. 4C) and did not express detectable ERAP2 protein (Fig. 4G). Two samples in the correlation analysis also displayed this mRNA expression pattern (rs2248374 AG, rs2910686 TT), as indicated in Fig. 3A.

### ERAP2 genotype affects expression level of related genes upon proinflammatory stimulation

**Fig. 5:**
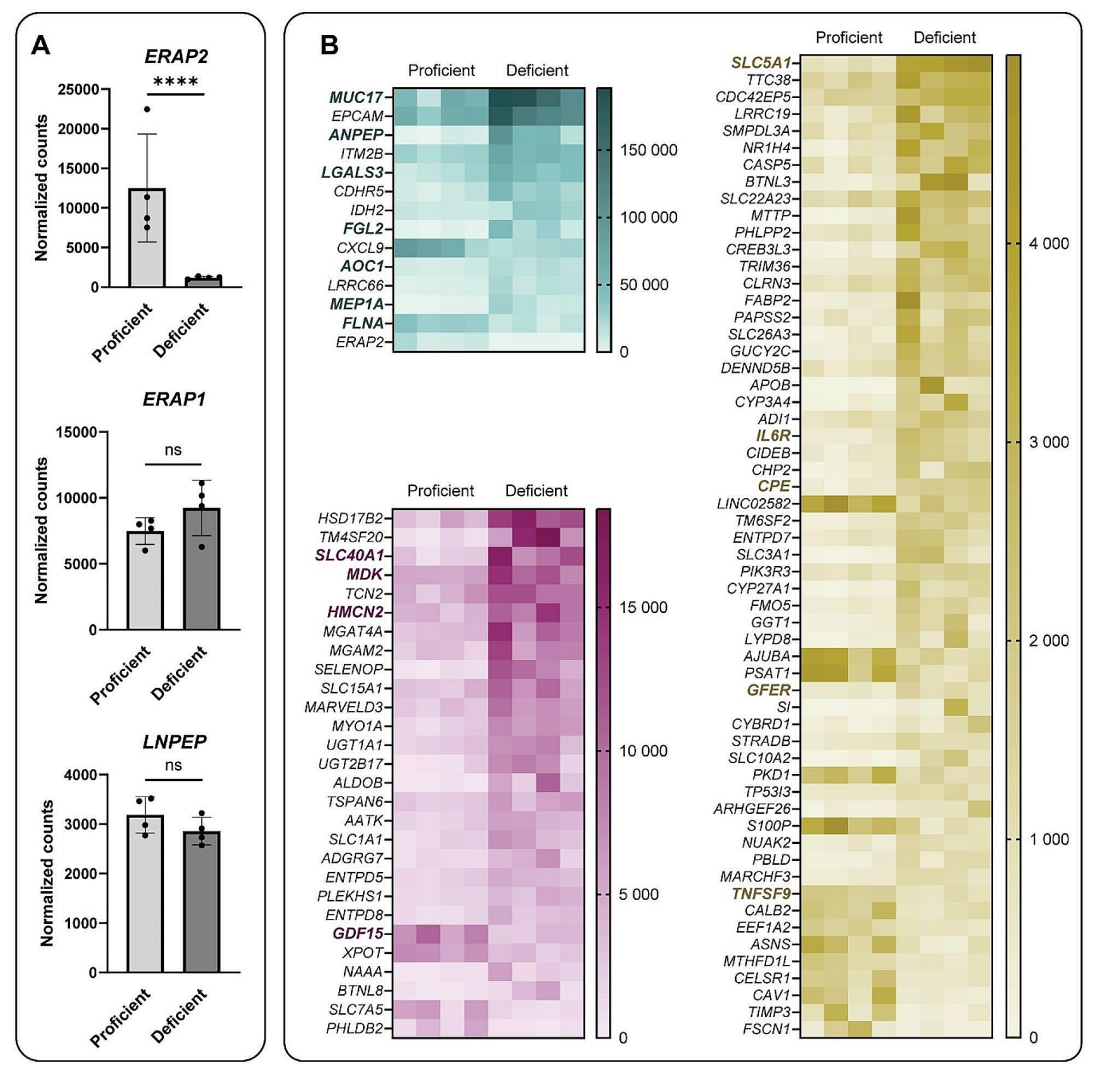
Gene expression in ERAP2-proficient and -deficient colonoids. (**A**) Expression levels of the oxytocinase subfamily of M1 aminopeptidases (ERAP2, ERAP1, LNPEP) in ERAP2-proficient and -deficient colonoids. (**B**) Heatmaps of top 100 differentially expressed genes meeting the threshold of P value < 0.01, Log_2_ fold change > 1 or < -1, and mean count > 1000 (for the highest expressing group). Genes are grouped based on maximum expression level per gene; > 20 000 (turquoise), 5 000–20 000 (purple) and < 5000 (yellow). Highlighted genes are shown as individual results in Fig. 6

A total of 586 genes were differentially expressed in the contrast ERAP2-deficient vs. ERAP2-proficient colonoids (*P* < 0.05) 24 h post IFNγ stimulation (Additional File 4: Table S4). The ERAP2-deficient group includes donors genotyped as rs2910686 TT, whereas the ERAP2-proficient group includes donors genotyped as CC or CT. Data is available at GSE266208. Other members of the oxytocinase subfamily of M1 aminopeptidases, *LNPEP* and *ERAP1*, were not differentially expressed between the ERAP2-deficient and -proficient group (Fig. 5A). The top GO Process and GO Localization was *small molecule metabolic process* (GO:0044281) and *cell periphery* (GO:0071944), respectively. The former term encompasses chemical reactions and pathways that involve small molecules (non-encoded molecules of low molecular weight, e.g. monosaccharides), whereas the latter term encompasses proteins located to the plasma membrane, cell cortex and any external encapsulating structures. Reports of enriched GO *Molecular Functions*, *Processes*, and *Localizations* are provided in Additional File 5: Table S5. To facilitate functional interpretation, a subset of genes was identified using a more stringent cut-off at P value < 0.01, Log_2_ fold change > 1 or < -1, and mean count > 1000 (for the highest expressing group), resulting in 100 genes (Fig. 5B).

**Fig. 6:**
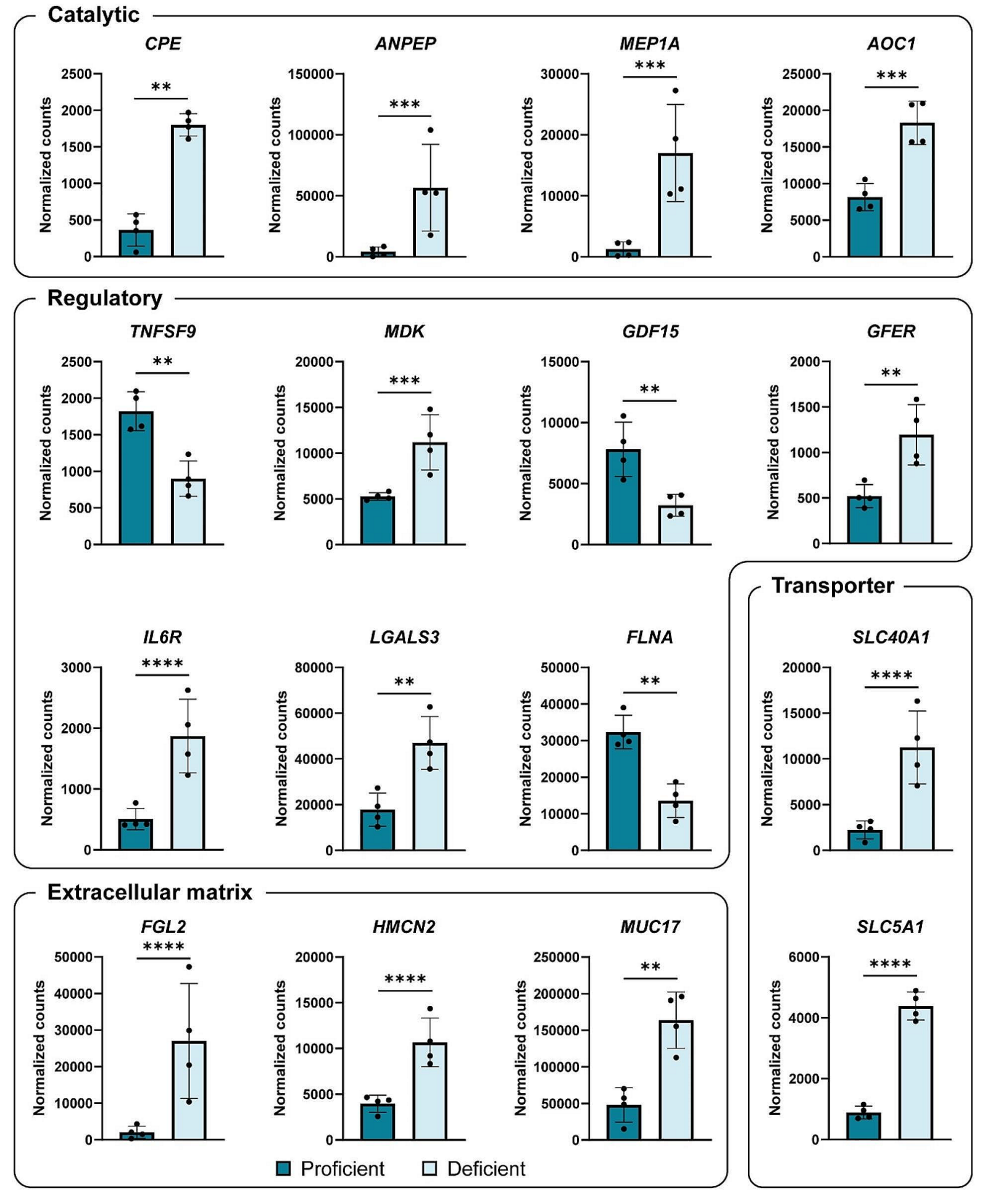
Subset of differentially expressed genes between ERAP2-proficient and -deficient groups meeting the set cut-off (P value < 0.01, Log2 fold change > 1 or < -1, and mean count > 1000 for the highest expressing group). The presented genes encode proteins with the following molecular function: catalytic activity (*AOC1*, *CPE*, *ANPEP* and *MEP1A*), regulator activity *(TNFSF9*, *MDK*, *GDF15*, *ILR6A*, *LGALS3* and *FLNA*), transmembrane transporter activity *(SLC40A1* and *SLC5A1*), and extracellular matrix structural constituents (*FGL2*, *HMCN2*, and *MUC17*). Expression presented as normalized counts

To enhance our understanding of differentially expressed genes meeting the set cut-off, we used GO *Molecular Functions* for grouping of genes. Almost half (46/100) of the thresholded genes encode proteins with catalytic activity (GO:0003824), including diamine oxidase (*AOC1*) and the peptidases carboxypeptidase H (*CPE*), aminopeptidase N (*ANPEP*), and meprin α (*MEP1A*). Close to 20% (19/100) encode proteins with regulator activity (GO:0098772), including CD137 ligand (*TNFSF9*), midkine (*MDK*), Growth/Differentiation Factor 15 (*GDF15*), Augmenter Of Liver Regeneration (*GFER*), Interleukin 6 receptor (*IL6RA*), galectin-3 (*LGALS3*) and filamin A (*FLNA)*. Seventeen genes encode proteins involved in transmembrane transporter activity (GO: 0022857), including iron exporter Ferroportin 1 (*SLC40A1*) and Sodium/Glucose Cotransporter 1 (*SLC5A1*). Regulated extracellular matrix structural constituents (GO:0005201) were Fibrinogen like 2 (*FGL2*), Hemicentin 2 (*HMCN2*), Mucin 17 (*MUC17*). Above-mentioned genes are presented in Fig. 6.

## Discussion

In this work, we have shown that ERAP2 is upregulated during colonic IBD inflammation, and that expression proficiency depends on genotype. We have further explored how pro-inflammatory stimuli regulate ERAP2 expression in the epithelium. We also confirm that additional mechanisms besides rs2248374 genotype can lead to ERAP2 deficiency. To gain insight into epithelial effects of ERAP2 presence under inflammatory conditions, we performed whole genome differential expression analysis on ERAP2-deficient vs. ERAP2-proficient colonoids post IFNγ stimulation (24 h), identifying 586 differentially expressed genes.

We believe that a better understanding of how genetic variation affects inflammatory responses is central to improving disease prognosis and treatment response of immune-mediated diseases. This is also reflected in the recent publication on “Challenges in IBD Research 2024: Preclinical Human IBD Mechanisms” initiated by The Crohn’s and Colitis foundation, where this is presented as a central research gap that needs to be addressed [27]. Our work focuses on dissecting the differences in inflammatory responses in ERAP2-proficient and -deficient epithelium and point to molecular mechanisms that are altered in inflamed epithelium devoid of ERAP2. A recent study on treatment response to the IL-17 A antibody Secukinumab in psoriasis patients demonstrated that ERAP2 deficient patients (as indicated by carrying the rs2248374 G/G genotype) had a 6-fold increased risk of treatment failure compared to ERAP2 proficient patients [28].

Selection for functional ERAP2 is thought to have arisen as a protective mechanism towards infection, supported by several recent studies [7, 8]. However, high expression of ERAP2 is in general viewed as unfavorable in the context of inflammation, with multiple SNPs in the genomic neighborhood of ERAP2 associated with inflammatory diseases, including IBD (especially Crohn’s Disease), ankylosing spondylitis, birdshot chorioretinopathy, psoriasis, preeclampsia, and hypertension [9–11]. Thus, ERAP2 presence seems to have opposed outcomes depending on environmental and biological state. Our paired genotype-gene expression data from colonic pinch biopsies show that ERAP2 expression is increased in the inflamed mucosa of IBD patients compared to uninflamed individuals, an observation that is masked when genotype is not taken into consideration. Although not explicitly discussed, the differential expression of ERAP2 in IBD is detectable in a few previously published data sets [29, 30]. The induction of ERAP2 by IFNγ along with the rest of the antigen presenting machinery has previously been shown by various studies [26]. In colonoids, we found ERAP2 mRNA and protein to be induced 24 h after IFNγ stimulation.

In our analysis, we surprisingly saw ERAP2 deficiency in individuals carrying the rs2248374-A variant. Most sources view this haplotype to be ERAP2-proficient, whereas the G allele results in a truncated mRNA degraded through nonsense-mediated decay [6]. In the colonoid experiments, we found Donor 8 (rs2248374 AA/rs2910686 TT, Fig. 4G) to be ERAP2-deficient, an unexpected observation due to its rs2248374 genotype. We also observed low *ERAP2* expression in two individuals included in the paired gene expression and genotyping data, despite being heterozygous at rs2248374 (Fig. 3A). SNPs rs2910686 and rs2248374 are in tight, but not perfect, LD in the European population (R^2^ = 0.805), with 5.4% of the population displaying the rs2910686-T/rs2248374-A haplotype [31]. The rs2910686 SNP is an intron variant located between exon 18 and 19 in *ERAP2* and has previously been associated with IBD [32].

ERAP2 is a highly polymorphic gene, with numerous SNPs proposed as expression quantitative trait loci. To get conclusive evidence for which SNP(s) or mechanisms that are causative for our observation of full-length ERAP2 loss, one would need to correlate gene expression and de novo sequence a large cohort. Venema et al. recently confirmed that ERAP2 expression is critically dependent on SNP rs2248374 [33]. Moreover, they show that disease risk SNPs downstream of ERAP2 influence expression independent of rs2248374, through alteration of enhancer-promoter interactions of ERAP2. This suggests that ERAP2 may be implicated in inflammatory diseases not only by its presence or absence, but also by its expression levels [33].

Functional interpretation of differentially expressed genes is not trivial, as many genes are pleiotropic in nature. Thus, our interpretation cannot conclude on the role of all the differentially expressed genes in this setting. However, we identified a subset of genes we believe to be of interest, given our identification of these as differentially expressed between ERAP2-deficient and proficient colonic organoids upon pro-inflammatory stimulation. We also performed an enrichment analysis, identifying GO *Processes*, *Molecular Functions* and *Localization* terms enriched among differentially expressed genes.

For functional interpretation of differentially expressed genes, a set of 100 genes was explored. Among these were *CPE*, *ANPEP* and *MEP1A*, encoding the peptidases carboxypeptidase E, aminopeptidase N (APN, also known as CD13) and Meprin α, respectively, all of which were more highly expressed in ERAP2-deficient organoids. CPE and APN generate bioactive proteins through processing of pro-peptides, and both proteins can be found in either membrane-bound or soluble form [34, 35]. CPE is specifically expressed in enteroendocrine cells, a cell type known to be dysregulated in IBD [36]. Mice deficient of CPE have been demonstrated to be more prone to DSS-induced colitis, and the protein thus been suggested to possess immunosuppressive effects in the intestinal mucosa [37]. As a multifunctional protein, APN is involved in the processing of various proteins and peptides, including hormones, cytokines, extracellular matrix proteins and antigens [34]. The protein also acts as a viral entry receptor [38]. We have previously found *CPE* to be upregulated, and *ANPEP* to be downregulated, in inflamed colonic epithelium isolated from IBD patients [39]. Meprin α is a secreted protease involved in extracellular matrix remodeling, receptor shedding and cytokine processing, and thus plays a regulatory role in inflammatory responses [40, 41]. *MEP1A* have previously been implicated as a susceptibility gene for IBD, where decreased expression was associated with intestinal inflammation in both IBD patients and animal models of IBD [42].

*AOC1*, *FLNA* and *GFER* all encode proteins suggested to be involved in tissue regeneration and permeability [43–45]. *AOC1* encodes diamine oxidase, which catalyzes the oxidative deamination of polyamines typically formed in excess during tissue growth and regeneration [46]. Dysregulation of the polyamine metabolism is implicated in several diseases, and both polyamines and their derivatives are involved in a wide array of molecular processes [47]. *FLNA* encodes Filamin A, an actin-binding protein active in the formation and rearrangement of actin cytoskeleton, as well as anchoring of a variety of proteins to actin filaments [44]. *GFER* encodes the protein “Augmenter of liver regeneration”, a growth factor that is most actively studied in liver epithelium but that also has a role in many other tissues, amongst them colon [48].

*MDK*, *GDF15*, *LGALS3* encode midkine, growth/differentiation factor 15, and Galectin 3. Midkine promotes production of inflammatory mediators and tissue infiltration of immune cells and is thus regarded as a pro-inflammatory cytokine [49]. Midkine has also been shown to have antibacterial activity in vitro [50]. Midkine is highly expressed during active IBD and has even been reported as overexpressed during quiescent CD [51]. We have previously shown that midkine is upregulated in the inflamed epithelium of IBD patients [39].

Macrophage inhibitory cytokine 1 (MIC1, *GDF15*), is a stress-induced cytokine belonging to the transforming growth factor-beta superfamily [52]. We have previously found *GDF15* to be upregulated in inflamed colonic epithelium isolated from IBD patients [39]. To our knowledge, GDF15 has not previously been associated with IBD pathogenesis.

Galectin 3, encoded by LGALS3, is a galactose-binding lectin involved in regulation of cell growth and apoptosis, as well as both adaptive and innate immunity [53, 54]. In IBD research, Galectin 3 expression has both been reported as decreased in intestinal mucosa during active disease and increased in blood serum of IBD patients both in remission and active disease [55, 56].

TNFSF9 and IL6R both encode proteins linked to immune modulation [57, 58]. TNFSF9 encodes 4-1BBL, a trans-membrane ligand for the T-cell co-stimulatory receptor protein 4-1BB. 4-1BBL’s interaction with immune cells through 4-1BB can be both pro- and anti-inflammatory though a wide array of interactions [57]. Interestingly, it has been demonstrated that 4-1BBL can sustain a pro-inflammatory immune response independently of 4-1BB, through interaction with Toll-like receptors and subsequent induction of pro-inflammatory TNFα production [59]. IL6R encodes a receptor for IL6 and exists both in a membrane bound and soluble form [60]. In IBD, increased IL6R expression and detectable soluble IL6R in peripheral blood is correlated with disease activity [61].

Within the list of 100 highly expressed and differentially regulated genes, seventeen were identified as involved in transporter activity. This included the genes *SLC40A1* (Ferroportin 1) and *SLC5A1* (Na/Glucose Cotransporter 1). In our previous work, we found upregulation of both *SLC40A1* and *SLC5A1* in the colonic epithelium of active IBD, and increased levels of Ferroportin 1 protein have been demonstrated in UC [62]. Together with the iron-regulatory hormone hepcidin, Ferroportin 1 controls systemic iron homeostasis in vertebrates, a process known to be influenced by inflammation [63]. Of note, iron deficiency anemia is one the most prevalent extraintestinal manifestations of IBD [64]. Na/Glucose Cotransporter 1, also known as SGLT1, is the primary mediator of intestinal glucose import [65]. While predominantly expressed in the small intestine, we have shown that *SLC5A1* is upregulated in colonic epithelium from IBD patients [39].

Three genes denoted as structural constituents of the extracellular matrix where differentially expressed between proficient and deficient donors, namely *HMCN2* (Hemicentin 2), *MUC17* (Mucin 17) and *FGL2* (Fibrinogen-like protein 2), all three being higher expressed in deficient donors. Hemicentin 2 is an extracellular matrix protein highly conserved across species, whose function remains largely unknown. However, animal studies have demonstrated that hemicentins are involved in forming extracellular matrix structure, and that Hemicentin 2 is expressed in cells of epithelial origin [66]. Mucin 17 is membrane-anchored mucin that is part of the protective intestinal glycocalyx lining the epithelium [67]. As with many other barrier proteins, Mucin 17 is reported to be dysregulated during IBD [68]. Fibrinogen-like protein 2 is an immunomodulatory protein that exists in either soluble or membrane-associated form, with epithelial cells capable of expressing the latter [69]. Dysregulation of FGL2 is associated with several inflammatory diseases, including IBD [70]. Furthermore, FGL2 deficient mice develop more severe inflammation upon DSS treatment [71].

It has previously been shown that certain variants correlate with an inverse expression of ERAP1 and ERAP2 [72, 73]. In our analysis of ERAP2-deficient and -proficient colonoids, there was no significant difference in mRNA expression of either *ERAP1* or *LNPEP*, the other members of the oxytocinase subgroup of M1 aminopeptidases.

Our work relies on RNA-seq data to show differences in the epithelial inflammatory response in ERAP2-deficient and -proficient colonoids. While our analysis highlights differences in RNA expression between the two groups, we do not demonstrate how these differences translate to functional consequences. Follow-up studies should focus on better characterization of how organoids differ in i.e. growth and differentiation. Further studies could also shift focus towards clinical data and stratify patient cohorts based on ERAP2 genotype, searching for clinical or treatment-response consequences of ERAP2 deficiency/proficiency.

We utilized patient-derived organoids in our experiments, a method that has some inherit limitations. As organoids are known to be heterogeneous in terms of stemness and differentiation potential between donors, we cannot exclude that some of the observed expressional differences between ERAP2-proficient and deficient donors may be attributed to variances in cellular composition unrelated to genotype. Neither can we exclude that the presence or absence of ERAP2 influences the cell composition. While donor-specific differences cannot be completely avoided, several steps were taken to minimize technical variation. We isolated both protein and RNA from cells grown in four and three wells, respectively, from each donor to minimize the risk of differences induced by technical variation. Furthermore, one donor from each group (proficient/deficient) was run in parallel, ensuring equal media composition for each experimental setup.

In our study the colonoids were grown in an atmosphere with 2% oxygen. Oxygen levels of the intestine vary along its length, and along the crypt-lumen axis. There is a drastic decrease in oxygen levels across the intestinal wall, dropping to PO_2_ levels approaching 0.4 kPa (∼ 0.5%) in the healthy colonic lumen, and even lower during inflammation [74]. Consequently, intestinal epithelial cells naturally exist in an environment of physiological hypoxia [75]. Our lab has previously shown that cultivation at 2% oxygen improves colonoid growth and differentiation, and that cultivation at 20% oxygen renders colonoids hyperresponsive to proinflammatory stimulation [25]. To better mimic in vivo conditions, it is now standard practice in our laboratory to cultivate colonoids at 2% oxygen, rather than the traditional 20%.

## Conclusions

We find *ERAP2* expression to be upregulated in the colonic mucosa of inflamed IBD patients. In addition, we also confirm that rs2248374 genotype is not the sole determinant of ERAP2 expression. Our study provides a set of differentially expressed genes between ERAP2-deficient and ERAP2-proficient colonoids post IFNγ stimulation, shedding light upon potential pathways affected by ERAP2 presence or absence during inflammatory encounter. The fact that every fourth individual lacks full-length ERAP2 indicates the protein to be dispensable for normal MHCI presentation, and its biological relevance has thus been questioned. This study lends support to the hypothesis that epithelial ERAP2 has pleiotropic effects beyond MHCI presentation. Our dataset serves as a resource for future studies aiming to reveal epithelial ERAP2 significance in inflammation and beyond peptide trimming.

## Electronic supplementary material

Below is the link to the electronic supplementary material.

Supplementary Material 1

Supplementary Material 2

Supplementary Material 3

Supplementary Material 4

Supplementary Material 5

## Data Availability

Data from RNA-sequencing is available at GSE266208.

## Abbreviations

CD: Crohn’s Disease
CGM: Complete growth medium
DM: Differentiation medium
ERAP: Endoplasmic reticulum aminopeptidase
FGL2: Fibrinogen Like 2
FLNA: Filamin A
GDF15: Growth Differentiation Factor 15
GFER: Growth Factor Erv1-Like
HMCN2: Hemicentin 2
IBD: Inflammatory bowel disease
IL6R: Interleukin 6 Receptor
LGALS3: Lectin, Galactoside-Binding, Soluble 3
MDK: Midkine
MHCI: Major histocompatibility complex class I
MUC17: Mucin 17
RNA-Seq: RNA-Sequencing
SLC40A1: Solute Carrier Family 40 Member 1
SLC5A1: Solute Carrier Family 5 Member 1
SNP: Single nucleotide polymorphism
TNFSF9: TNF Superfamily Member 9
UC: Ulcerative Colitis
