# Correction to: The effect of rs2910686 on ERAP2 expression in IBD and epithelial inflammatory response

**DOI:** 10.1186/s12967-024-05645-2

**Published:** 2024-09-20

**Authors:** Siri Sæterstad, Ann Elisabeth Østvik, Marianne Doré Hansen, Torunn Bruland, Atle van Beelen Granlund

**Affiliations:** 1 Department of Clinical and Molecular Medicine, Norwegian University of Science and Technology (NTNU), Trondheim, Norway; 2 Department of Gastroenterology and Hepatology, Clinic of Medicine, St. Olav’s University Hospital, Trondheim, Norway; 3 Clinic of Laboratory Medicine, St. Olav’s University Hospital, Trondheim, Norway; 4 Department of Pathology, St. Olav’s University Hospital, Trondheim, Norway; 5 Centre of Molecular Inflammation Research, Norwegian University of Science and Technology (NTNU), Trondheim, Norway

Following publication of the original article [1], we have been notified that supplementary file 6 was not supposed to be published as it contained information on response to reviewers.

The original article was updated.
