# Supplementary material for: The effect of rs2910686 on ERAP2 expression in IBD and epithelial inflammatory response
Source: J Transl Med. 2024 Aug 9;22:750. doi: 10.1186/s12967-024-05532-w (PMC11316291; doi:10.1186/s12967-024-05532-w)
Supplement: Supplementary file 1 — Supplementary Material 1 [file 12967_2024_5532_MOESM1_ESM.pdf]

Table S1: Composition of Minigut B.

	Concentration	Catalog	Manufacturer
Advanced DMEM/F-12		12634028	Gibco
BSA	1% (w/v)	A7906	Sigma-Aldrich
GlutaMAX™ 100X	1X	35050061	Gibco
N-2 Supplement 100X	1X	17502001	Gibco
B-27 Supplement 50X	1X	17504044	Gibco
HEPES 1M	10mM	15630080	Gibco
Penicillin-Streptomycin	100 U/mL	15140122	Gibco

Table S2: Composition of complete growth medium.

	Concentration	Cell line/catalog	Manufacturer
Wnt-3A CM*	50%	CRL-2647	ATCC
Minigut B	30%	-	-
R-spondin 1 CM*	20%	AMS.RSPO1-CELLS	AMSBIO
Nicotinamide	1221.2 µg/mL	N0636	Sigma-Aldrich
N-Acetyl-L-cysteine	163.2 µg/mL	A9165	Sigma-Aldrich
Noggin	0.1 µg/mL	120–10C	PeproTech
A-83-01	0.211 µg/mL	SML0788	Sigma-Aldrich
SB202190	3.314 µg/mL	S7067	Sigma-Aldrich
EGF	0.05 µg/mL	AF-100-15	PeproTech
Gastrin	0.021 µg/mL	G9145	Sigma-Aldrich
Y-27632**	3.203 µg/mL	72304	STEMCELL Technologies

*CM; Conditioned media

**Day one and three only

Table S3: Composition of differentiation medium.

	Concentration	Cell line/catalog	Manufacturer
Wnt-3A CM*	5%	CRL-2647	ATCC
Minigut B	75%	-	-
R-spondin 1 CM*	20%	AMS.RSPO1-CELLS	AMSBIO
N-Acetyl-L-cysteine	163.2 µg/mL	A9165	Sigma-Aldrich
Noggin	0.1 µg/mL	120–10C	PeproTech
A-83-01**	0.211 µg/mL	SML0788	Sigma-Aldrich
EGF	0.05 µg/mL	AF-100-15	PeproTech
Gastrin	0.021 µg/mL	G9145	Sigma-Aldrich
DAPT	4.325 ug/mL	2634	Tocris

*CM; Conditioned media

**Withdrawn the day of stimulation
