# Supplementary material for: The effect of rs2910686 on ERAP2 expression in IBD and epithelial inflammatory response
Source: J Transl Med. 2024 Aug 9;22:750. doi: 10.1186/s12967-024-05532-w (PMC11316291; doi:10.1186/s12967-024-05532-w)
Supplement: Supplementary file 2 — Supplementary Material 2 [file 12967_2024_5532_MOESM2_ESM.pdf]

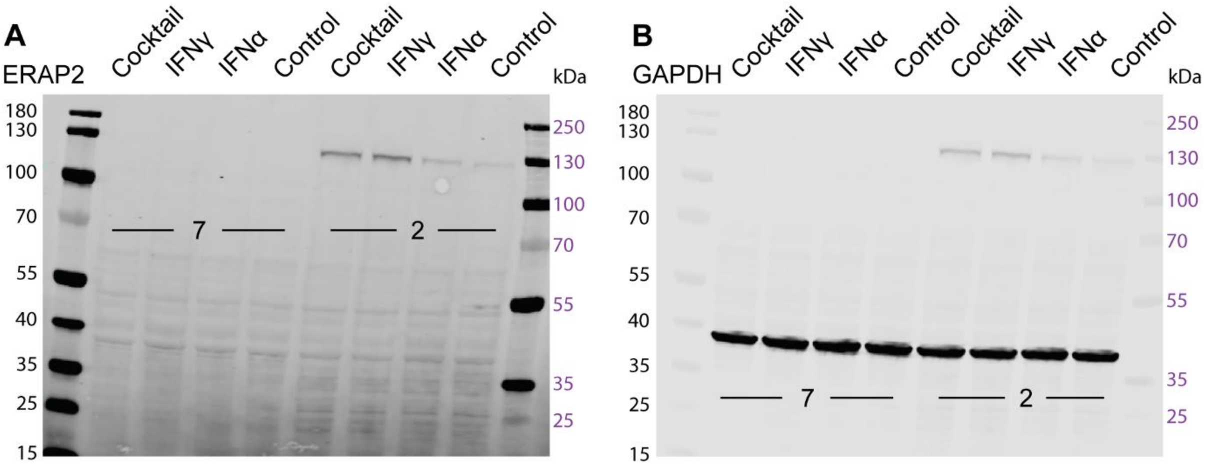

Figure S1: Uncropped membrane for the blot included in Figure 3A for donor 7 (ERAP2-deficient) and 2 (ERAP2-proficient), for **A)** ERAP2 and **B)** GAPDH. Conditions indicated for each lane (proinflammatory cocktail, IFNɣ, IFNα, control). PageRuler (black) and PageRuler Plus (purple) used as protein ladders, kDa indicated. The primary antibody used to detect ERAP2 is Goat anti-Human, and the secondary antibody used to detect GAPDH is Donkey anti-Goat, resulting in ERAP2 being visible in B).
