# Supplementary material for: The effect of rs2910686 on ERAP2 expression in IBD and epithelial inflammatory response
Source: J Transl Med. 2024 Aug 9;22:750. doi: 10.1186/s12967-024-05532-w (PMC11316291; doi:10.1186/s12967-024-05532-w)
Supplement: Supplementary file 3 — Supplementary Material 3 [file 12967_2024_5532_MOESM3_ESM.pdf]

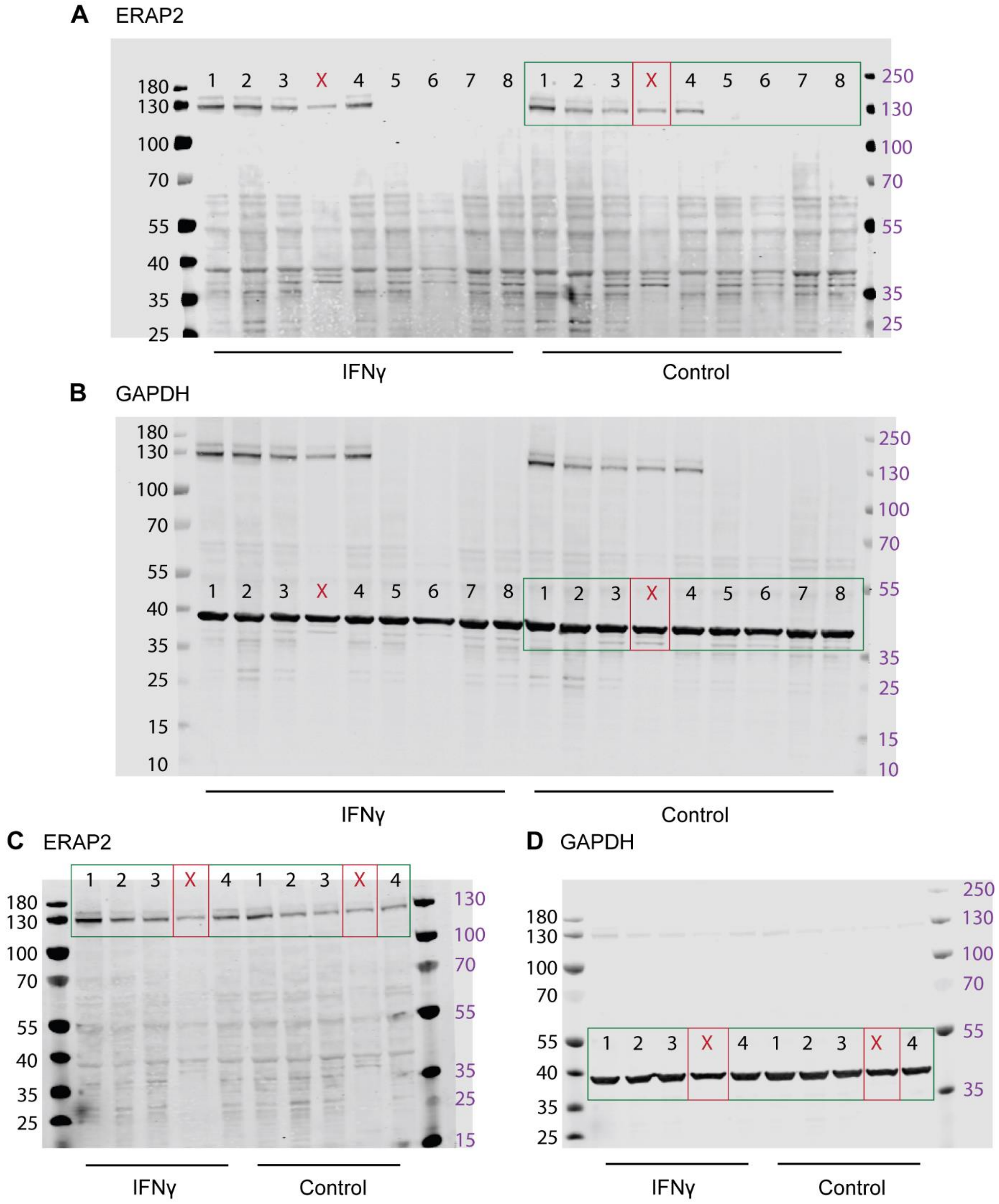

Figure S2: Uncropped membranes for blots included in Figure 3D and 3G. Donors (1-8) and condition (IFNɣ/control) indicated for each lane. PageRuler (black) and PageRuler Plus (purple) used as protein ladders, kDa indicated. Green boxes indicate included samples, red boxes indicate excluded samples (donor X). Blots used in Figure 3G include **A)** ERAP2 and **B)** GAPDH, 35µg protein loaded. Blot used in Figure 3D include **C)** ERAP2 and **D)** GAPDH, 22µg protein loaded. The primary antibody used to detect ERAP2 is Goat anti-Human, and the secondary antibody used to detect GAPDH is Donkey anti-Goat, resulting in ERAP2 being visible in B) and D).
